# The Popular and Medical Uses of Narcotics

**Published:** 1919-01-25

**Authors:** 


### The Hospital

The Workers' Newspaper of Administrative Medicine and Institutional Life, Administration, National Insurance and Health.

No. 1703, Vol. LXV. SATURDAY, JANUARY 25, 1919. PRICE TWOPENCE

### THE POPULAR AND MEDICAL USES OF NARCOTICS.

If, as we may assume to be the case, the news which reaches us from the other side of the Atlantic is correct, we are about to witness, and on a tremendous scale, a highly interesting and important social experiment. For a number of years certain of the States of the American Union have professed through legislative enactment the policy of a total prohibition of alcohol in their several areas, and not a few travellers have told us of various ingenious devices by which the law could be successfully evaded. But these local restrictions fade into insignificance before the announcement that in the whole of the territory of the United States alcoholic beverages are to come under the ban of the law, and are neither to be bought, nor sold, nor manufactured, nor imported. The programme is a gigantic one. It embraces a country which stretches from ocean to ocean and affects a hundred million of people. Moreover, it touches a habit which has behind it the tradition of generations, and in the practice of which men, whether wisely or unwisely, have sought not only æsthetic pleasure, but also solace and inspiration. Like other habits, the taking of alcohol is, of course, capable of abuse, and from this abuse manifest personal and social evils have arisen. The practical issue at the moment seems to be whether these evils are so extreme, and so resistant to all other remedies, as to justify such an invasion of the liberty of the individual citizen as is involved in the practice of absolute and complete legal prohibition. The decision of the United States clearly affirms this proposition, and the operation of such a decision will be watched with a high degree of interest. As is inevitable in connection with a question which has involved so much controversy, there are eager anticipations both in the one direction and the other. While some of the prophets assure us that a millennium is at hand, and that an alcohol-free people is about to carry off all the prizes in the economic struggles of the immediate future, others are convinced that the restrictions will stimulate methods of evading the law, will encourage perhaps the worst form of indulgence, namely, secret drinking, and will both bring the law into contempt and weaken the morale of the people. For our own part we make no pretence to disentangle the future, and we have no right to criticise a decision which in its immediate application is purely a domestic question for the American people. All the same we shall watch the upshot with great interest and with all good will. The effect, whatever it be, will not be confined to the Western hemisphere, and it is hard to think it will be confined to one habit. Democracy, if we may judge some of its present voices, has no more patience with manifestations of individual choice and habit than had some of our fallen autocrats. Has even the drill-sergeant ever reached the courage to fix the dietetic habits of the private citizen? Strange things have been done in the name of "Liberty," and evidently the chapter is not yet closed.

Meanwhile, close at home we have gloomy evidences of the completeness with which the severest regulations fail before the efforts of resolute and unscrupulous people; and what comes to the surface is only a fraction of what actually exists. The general regulations which govern the sale of poisonous alkaloids have been immensely strengthened by Orders issued under the authority of the Defence of the Realm Act, yet, as is now displayed in open court, cocaine is bought and sold on a large scale, and nothing but a chance or accident leads to discovery. In how many similar transactions, we wonder, has the secret remained unrevealed? The fact is that, granted the existence of appetite and of money, the law cannot devise a plan which will form an absolute barrier to people set on self-gratification on the one hand and on financial profit on the other. The mischief may be limited and kept under restraint, but its complete suppression is almost always impossible. Further, it has to be admitted that to certain types of mind secrecy and defiance of the law contribute an additional stimulus, and the pleasures they gain from self-drugging are the sweeter because they are stolen. No one acquainted with the facts would advocate a relaxation of the existing restrictions, but even these, severe as they are, are not wholly successful when set in opposition to perverted appetite.

The public advertisement, through the courts and otherwise, of the prevalence of the drug habit has some reaction on the medical profession. Naturally it leads to a growing disinclination to the use of narcotic drugs, for no medical practitioner would care to be responsible for an intervention which could be regarded as the beginning of a pernicious habit. At the same time it must be remembered that these drugs have their uses and values, and cannot therefore be excluded from sane and helpful medical practice. The risk of habit may have to be faced, and while an unhappy development, when it occurs, experience shows that the event is not a frequent one. Thousands of patients are helped back to health by drugs which are capable of abuse if committed to irresponsible hands. The safety is in keeping such medicines under medical direction. When this is escaped quantitative control is lost, and it is here that the great mischief of increasing doses becomes possible. Members of the profession know well many chronic sufferers whose lives are rendered comfortable and even useful by the administration over long periods of such a drug as opium, and to take away this solace because it may in some instances be abused would be but a feeble policy. The lesson is that, while care should be exercised before such a practice is selected, the physician should not shrink from responsibility when he is convinced that the use of narcotics is favourable to the patient's welfare.

